# Epidemiological description of and response to a large yellow fever outbreak in Edo state Nigeria, September 2018 - January 2019

**DOI:** 10.1186/s12889-022-14043-6

**Published:** 2022-08-30

**Authors:** E. Nwachukwu William, John Oladejo, Chinenye Mary Ofoegbunam, Chimezie Anueyiagu, Festus Dogunro, Sandra Okwudili Etiki, Botson Iliya Dachung, Celestina Obiekea, Bukola Aderoju, Kayode Akanbi, Idayat Temitope Adeyemi, Gboyega Adekunle Famokun, Obi Emelife, Irowa Williams Osamwonyi, Chinwe Lucia Ochu, Alice Abiode, Faith Ireye, Martins Chukwuji, Oladipupo Ipadeola, Musa Saiki, Ifeanyi Okudo, Dorathy Nwodo, Joseph Avuwa Oteri, Elsie Ilori, Nwando Mba, Chikwe Ihekweazu

**Affiliations:** 1 Nigeria Centre for Disease Control, Plot 800 Ebitu Ukiwe Street Jabi, Abuja, Nigeria; 2 National Arbovirus and Vector Research Centre, Abuja, Enugu State Nigeria Nigeria; 3 Nigeria Field Epidemiology and Laboratory Training Programme, Abuja, Nigeria; 4 National Primary Healthcare Development Agency, Abuja, Nigeria; 5 Ministry of Health, Benin City, Edo State Nigeria; 6 World Health Organization, Nigeria Office, Abuja, Nigeria; 7 University of Maryland Baltimore, Nigeria Country Office, Abuja, Nigeria

**Keywords:** Yellow fever, Outbreak, VPD, Edo-state

## Abstract

**Background:**

Edo State Surveillance Unit observed the emergence of a disease with “no clear-cut-diagnosis”, which affected peri-urban Local Government Areas (LGAs) from September 6 to November 1, 2018. On notification, the Nigeria Centre for Disease Control deployed a Rapid Response Team (RRT) to support outbreak investigation and response activities in the State. This study describes the epidemiology of and response to a large yellow fever (YF) outbreak in Edo State.

**Methods:**

A cross-sectional descriptive outbreak investigation of YF outbreak in Edo State. A suspected case of YF was defined as “Any person residing in Edo State with acute onset of fever and jaundice appearing within 14 days of onset of the first symptoms from September 2018 to January 2019”. Our response involved active case search in health facilities and communities, retrospective review of patients’ records, rapid risk assessment, entomological survey, rapid YF vaccination coverage assessment, blood sample collection, case management and risk communication. Descriptive data analysis using percentages, proportions, frequencies were made.

**Results:**

A total of 209 suspected cases were line-listed. Sixty-seven (67) confirmed in 12 LGAs with 15 deaths [Case fatality rate (CFR 22.4%)]. Among confirmed cases, median age was 24.8, (range 64 (1-64) years; Fifty-one (76.1%) were males; and only 13 (19.4%) had a history of YF vaccination. Vaccination coverage survey involving 241 children revealed low YF vaccine uptake, with 44.6% providing routine immunisation cards for sighting. Risk of YF transmission was 71.4%. Presence of Aedes with high-larval indices (House Index ≥5% and/or Breteau Index ≥20) were established in all the seven locations visited. YF reactive mass vaccination campaign was implemented.

**Conclusion:**

Edo State is one of the states in Nigeria with the highest burden of yellow fever. More males were affected among the confirmed. Major symptoms include fever, jaundice, weakness, and bleeding. Majority of surveillance performance indicators were above target. There is a high risk of transmission of the disease in the state. Low yellow fever vaccination coverage, and presence of yellow fever vectors (*Ae.aegypti, Ae.albopictus* and *Ae.simpsoni*) are responsible for cases in affected communities. Enhanced surveillance, improved laboratory sample management, reactive vaccination campaign, improved yellow fever case management and increased risk communication/awareness are very important mitigation strategies to be sustained in Edo state to prevent further spread and mortality from yellow fever.

**Supplementary Information:**

The online version contains supplementary material available at 10.1186/s12889-022-14043-6.

## Introduction

Yellow fever (YF) is an acute viral haemorrhagic disease characterised by fever, yellowness of the eyes, skin and urine caused by the yellow fever virus which belongs to the genus Flavivirus. It is a vector-borne (arbovirus) disease transmitted to man from the bites of infected *Aedes* mosquitoes. Humans and non-human primates are the reservoir hosts of yellow fever [1, 2]. There are three basic transmission cycles of yellow fever: i) the jungle (sylvatic) yellow fever virus transmission cycle, is between non-human primates (e.g. monkeys) and mosquito species; ii) The intermediate (savannah) cycle involves the transmission of the virus from infected mosquitoes to humans living or working in jungle border areas; iii) the urban cycle involves transmission of the virus between humans. Transmission occurs when an infected person from the jungle or savannah introduces the disease in human populations with low immunity for fellow fever [3, 4].

Yellow fever remains a public health problem, especially in Africa, despite the availability of an effective vaccine [5]. This is due to several factors including uncontrolled urbanisation with rapid encroachment into natural habitats of the yellow fever vector, low production capacity for yellow fever vaccines and limited enforcement of the International Health Regulations (IHR) by countries in the region [1, 2]. Yellow fever control is guided by the World Health Organization (WHO) coordinated “Elimination of Yellow Fever Epidemic (EYE) Strategy” with three strategic objectives namely: to protect at-risk populations (no epidemics), prevent international spread (no exportation) and contain outbreaks rapidly (no sustained transmission) [6].

The current cycle of yellow fever transmission in Nigeria was detected in September 2017, in Ifelodun Local Government Area (LGA) of Kwara State, 21 years after the last reported confirmed yellow fever case [7]. Since the onset of the outbreak, increasing numbers of cases with increasing geographic spread have been reported. From July 2017 to December 2018, a total of 163 confirmed cases in 46 LGAs in 17 states were reported from the Institut Pasteur (IP Dakar), Ninety deaths were reported (CFR = 2.2%) from all suspected cases and 31 deaths among confirmed cases (CFR = 19.0%) [8, 9].

As a major preventive measure, the yellow fever vaccine was introduced into routine immunization (RI) schedule nationwide in 2004 targeting children 9 months to 2 years. However, since the re-emergence of YF in 2017, yellow fever vaccinations have been accelerated through both preventive and reactive mass vaccination campaigns. As at first quarter of 2021, 11 international coordinating group for vaccine provision (ICG) requests were approved for YF reactive mass vaccination (RMVC) with about 15 million Nigerians vaccinated across 75 LGAs in 16 States. While 88,121,329 were vaccinated through preventive mass vaccination campaigns in 19 states. Planned phase PMVC schedule to cover all the states in Nigeria till 2025 [10].

The first recent confirmed case of yellow fever in Edo State was recorded in May 2018 of a 65-year-old woman from Etsako East LGA. The case presented with fever, jaundice and vomiting with no history of vaccination nor travel to yellow fever affected states. On November 14, 2018, the Epidemiology Unit of the Department of Disease Control, Edo State reported an observed incidence of a disease with “no clear-cut diagnosis” that required urgent attention, to the Nigeria Centre for Disease Control (NCDC). The cases presented with clinical signs and symptoms suggestive of a viral haemorrhagic disease, with dates of onset between September 6 and November 1, 2018. The cases were resident in four LGAs including Esan Central, Esan West, Owan East and Uhunmwode. The state has the highest Lassa fever (LF) burden in Nigeria [11]. Most LGAs with high LF burden were the LGAs that reported the strange disease. This led to a low index of suspicion of yellow fever in the affected communities which consequently increased the mortality experienced in the outbreak.

Preliminary investigations on the samples for Lassa fever at the Institute of Lassa Fever Research and Control (ILFRC), Irrua, Edo State were negative. Further investigations were done using IgM serology in Central Public Health Laboratory Lagos (CPHL) and metagenomic analysis at the African Center of Excellence for Genomics of Infectious Diseases (ACEGID), Redeemer’s University, Ede, Osun State**.** The results of these investigations were positive for yellow fever [12]. Following the notification of these cases, NCDC deployed a multi-disciplinary team to support the state’s response to the outbreak. The objectives of the deployment were to describe the re-emergence of yellow fever, assess the risk of a larger outbreak occurring, assess the determinants of the outbreak and define short, medium- and long-term control measures.

The aim of this study is to provide the descriptive epidemiology of and response to a large yellow fever (YF) outbreak in Edo State.

## Methods

### Study area/ study design

This is a cross-sectional descriptive outbreak investigation and response of yellow fever in Edo State, Nigeria as at January 2019. Edo State is one of the states in the South-South geo-political zone of the country with 18 LGAs [13]. The clusters of “cases of a strange illness” that initially affected four LGAs namely, Esan Central, Esan West, Owan East and Uhunmwode LGAs, later increased in both severity and geographic coverage extending to 12 LGAs between September 2018 and January 2019.

Advocacy visits were paid to key stakeholders to provide information about the presence and purpose of the team in the state and to obtain detailed information on the current situation and activities undergone. At the community level, advocacy visits were made to community leaders by the RRT detailing the nature and risks associated with the disease and preventive measures. Community leaders were sensitised on the case definition for yellow fever.

### Operational case definition

A modified standard case definition for YF from the integrated disease surveillance and response (IDSR) technical guidelines (2013) for Nigeria was adapted as the working case definition and utilised for the purpose of identifying suspected cases of YF residing in the communities in Edo State [14].

The study population included persons who met the case definitions of yellow fever as follows:

i. *Suspected Case:* Any person residing in Edo State with acute onset of fever, with jaundice appearing within 14 days of onset of the first symptoms with or without bleeding from September 1, 2018 to January 12, 2019.
ii. *Probable Case:* A suspected case whose sample was IgM positive / PCR positive/metagenomics positive in a national laboratory in the absence of YF vaccination within 30 days of onset of illness with an epidemiological link to a confirmed case or an outbreak and positive post-mortem liver histopathology.
iii. *Confirmed Case:* A probable case and the detection of YF-specific IgM, detection of a four-fold increase in YF IgM and/or IgG antibody titres between acute and convalescent serum samples, detection of YFV-specific neutralising antibodies at WHO Regional Reference Laboratory, Institut Pasteur.

Following the establishment of case definitions for the outbreak, the activities detailed below were subsequently carried out during the outbreak investigation:

i. Active case search

Active case search was done in line with the YF preparedness and response guideline and YF field investigation guide [15, 16] Active case search was conducted by the RRT at the health facilities and communities. For health facilities (HF), a retrospective review of HF records (registers/case notes) took place at the medical records, outpatient and inpatient and the laboratory sections from September 1, 2018 to January 12, 2019 was done. Patients who met the case definitions were added to a specific yellow fever outbreak line list.

Two approaches were used in the community active case search. Community leaders were sensitised on the case definition for yellow fever. The first approach was to assemble community members together in a place approved by the community leader where they were sensitised and examined for symptoms and signs of YF. The second approach was a house-to-house case search where the RRT visited every house in the community with an assigned community guard by the community leader.

Any person that met the case definitions for suspected case was added to a line-list and their blood sample collected. Detailed case investigation was carried out on all the confirmed cases. Human blood sample management.

The RRT facilitated sample management (collection, packaging, and transportation) as part of outbreak response activities. All suspected cases had 5mls of venous blood collected by the laboratory team. The samples stored in plain bottles were centrifuged at 500 g-1000 g for 5 min to obtain sera. The sera were collected into cryovial tube(s), stored at **+** 2 to **+** 8 °C or frozen at − 20 °C degrees Celsius (°C). These samples were triple packaged and shipped under good cold chain through a contracted courier company to the NCDC Central Public Health Laboratory (CPHL), Yaba, Lagos for IgM serology. Positive (presumptive positive) samples were sent to the World Health Organization (WHO) Regional Reference Laboratory, Institut Pasteur (IP) Dakar where both real-time polymerase chain reaction (RT-PCR) and plaque reduction neutralization test (PRNT) were used for final confirmation.

ii. Risk assessment

Risk assessment was done at the state level using a set of 14 criteria for the assessment:

Each criterium was given a maximum score of one and a minimum score zero (1 or 0): Total score was 14 while least score was 1. Earned score was divided by the total score and multiplied by 100. The percentage scores were graded thus: 70-100% is very high risk; 40-69% is moderate risk and below 40% is low risk. Data were collected using a pro forma, entered and analysed using Microsoft Excel.

In addition, a risk communication gap assessment to review existing documents and reports, inventory of existing communication materials and key informant interviews. Coordination and system strengthening, yellow fever jingle, media plan, training schedule for healthcare workers and community engagement were carried out.

iii. Verbal autopsy

Verbal Autopsy (VA) was used to estimate disease burden, mortality, and under-reporting of yellow fever as part of the National Yellow fever Outbreak Response Strategy. A case of VA was defined as “any death of a family member(s) who prior to death developed acute onset of fever and jaundice appearing within 14 days in a person who resided in Uhunmwode, Esan West, Esan Central and Owan West or any other LGAs within Edo State between September 1, 2019, to January 12, 2019” [7]. A questionnaire was used to collect data from family members. Any death in the community that met the case definition was included. However, all cases line listed in the VA were verified with the state surveillance data. Those already captured in the state surveillance data were excluded from the report.

iv. Entomological surveillance

An entomological survey was conducted in the first four LGAs to identify the presence of the yellow fever vectors. The approaches used to establish the presence of the vectors, *Aedes* mosquitoes, in the locations visited include *(i)* larval sampling, which was designed to collect immature stages (larvae and pupae) of the vectors. *(ii)* Ovitraps were designed to collect *Aedes* mosquito eggs. *(iii)* modified Human Landing Catch (mHLC), designed to collect adult mosquitoes Two types of adult collection traps were deployed: Biogents’-sentinel trap and CDC UV light trap [17–19].

xxii. Rapid Vaccination Coverage Assessment

Rapid Vaccination Coverage Assessment (RVC) was conducted in the four LGAs where the outbreak started to determine the yellow fever vaccination status of children 10 years and below in the community, as part of the national YF outbreak response strategy. A systematic sampling of alternate houses was used to identify those to be included. The assessment began where the RRT met with the community leader and the team subsequently moved in a clockwise direction. Children below the age of one and above 10 years were excluded. A living first-born child between 1 and 10 years in each house was studied until 10 children per settlement were identified and their caregivers interviewed. A caregiver at each selected house was asked for the history of yellow fever vaccination as well as documentary evidence in the routine immunisation (RI) cards to show that the child had YF vaccination. Sighting of the immunisation card and date of yellow fever vaccination was evident that the child received YF vaccination.

vi. Yellow fever reactive mass vaccination campaign

A request for YF reactive mass vaccination campaign was made through the International Coordinating Group (ICG) for vaccine provision. Upon approval by the ICG, pre-implementation and implementation microplans were developed. The campaign strategy was a fixed and temporary fixed post campaign strategy targeting the age groups of 9 months to 44 years (85% of total population).

vii. Data management and Analysis

Yellow fever specific investigation data tools were used for different activities, and these include.

Active case search: the yellow fever specific line-list in Excel template was used and analysed with Microsoft Excel software.

Verbal autopsy: data was collected using a structured-interviewer-administered questionnaire. Data was entered and analysed using Epi-Info software.

Risk assessment: Checklist was used for data collection and analyses with Microsoft Excel software.

Entomology: A customized excel template was used in collection of entomology data.

Rapid yellow fever vaccination coverage assessment: a checklist was used to collect data. Data was entered and analysed using Epi-Info software. RMVC data were collated and analysed using the yellow fever mass vaccination campaign database in Microsoft Excel.

All data analysis done were descriptive data analysis using percentages, proportions, and frequencies.

## Results

### Demographic characteristics of study participants

**Fig. 1:**
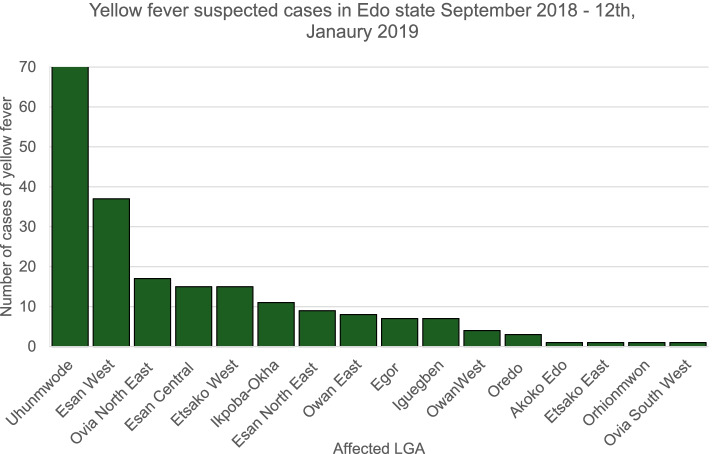
Suspected yellow fever cases in Edo State by LGAs September 2018 – January 2019

**Fig. 2:**
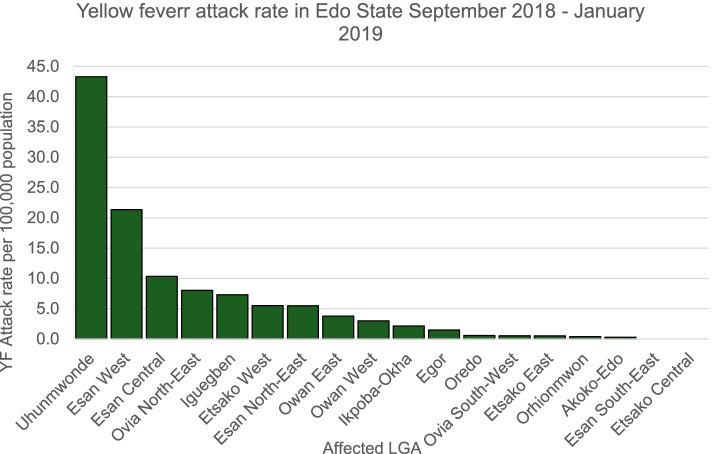
Yellow fever attack rate by LGA in Edo State September 2018 – January 2019

Two hundred and nine (209) suspected cases of YF were recorded from 16 LGAs during the active case search in the communities and the retrospective record review of data from health care facilities from September 1, 2018–January 12, 2019. Tables 1 and 2. The outbreak started from 4 peri-urban LGAs namely: Uhunmwode, Esan-central, Esan-West and Ovia North-East and these LGAs had both the highest number of cases and attack rate per 100,000 population. Seventy-two (34.4%) of the cases were from Uhunmwode LGA (Figs 1 and 2).

**Table 1:** Summary of demographic characteristics of suspected yellow fever cases in Edo State, September 2018–January 2019″

Demography and clinical characteristics of	Frequency (***N*** = 209) Percentage (%)
**Sex**	
Male	159 (76.1)
Female	50 (23.9)
**Age (years)**	
Age range	70 years (1 – 71 years)
Median age	20 years
**Affected LGAs**	
Uhunmwode	72 (34.4)
**Result**	
Presumptive positive	94 (45.0)
Confirmed	67 (32.1)
Male	51 (76.1)
Female	16 (23.9)
Not a case^a^	142 (67.9)
**Deaths**	
Suspected	25 (12.0)
Presumptive	17 (18.1)
Confirmed (IP Dakar)	15

^a^Not a case: All negative cases at both National and Regional Reference Laboratories excluding IP Dakar confirmed cases

**Table 2:** Classification of cases of yellow fever by LGA in Edo State from September 2018–January 2019

Yellow Fever Cases in Edo State September 2018 - January 2019			
Affected LGA	Number of Suspected Cases of YF (%)	Number of Presumptive Cases of YF (%)	Number of Confirmed Cases of YF (%)
Akoko Edo	1 (0.5)	1 (1.1)	1 (1.5)
Egor	7 (3.3)	0	0
Esan-Central	15 (7.2)	6 (6.4)	3 (4.5)
Esan North-East	9 (4.3)	5 (5.3)	4 (6.0)
Esan-West	37 (17.7)	19 (20.2)	14 (20.8)
Etsako East	1 (0.5)	0	0
Etsako West	15 7.2)	7 (7.4)	5 (7.5)
Iguegben	7 (3.3)	2 (2.2)	2 (3.0)
Ikpoba-Okha	11 (5.3)	7 (7.4)	4 (6.0)
Oredo	3 (1.4)	2 (2.2)	2 (3.0)
Orhionmwon	1 (0.5)	0	0
Ovia North-East	17 (8.2)	7 (7.4)	7 (10.4)
Ovia South-West	1 (0.5)	0	0
Owan-East	8 (3.8)	3 (3.2)	2 (3.0)
Owan-West	4 (1.9)	3 (3.2)	3 (4.5)
Uhunmwode	72 (34.4)	32 (34.0)	20 (29.8)
**Total**	**209 (100.0)**	**94 (100.0)**	**67 (100.0)**

**Fig. 3:**
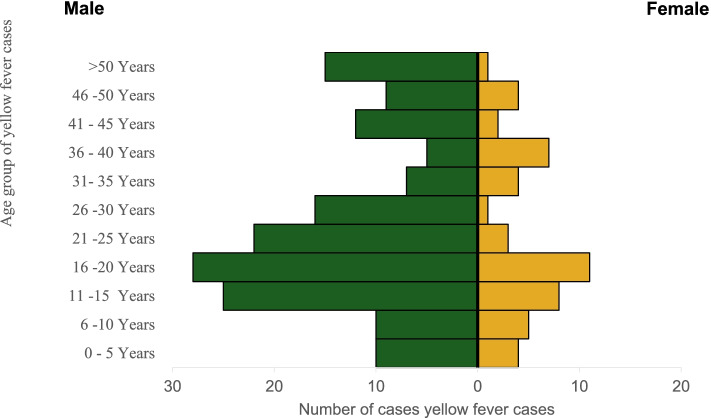
Age-sex distribution of yellow fever cases in Edo State September 2018 - January 2019

**Fig. 4:**
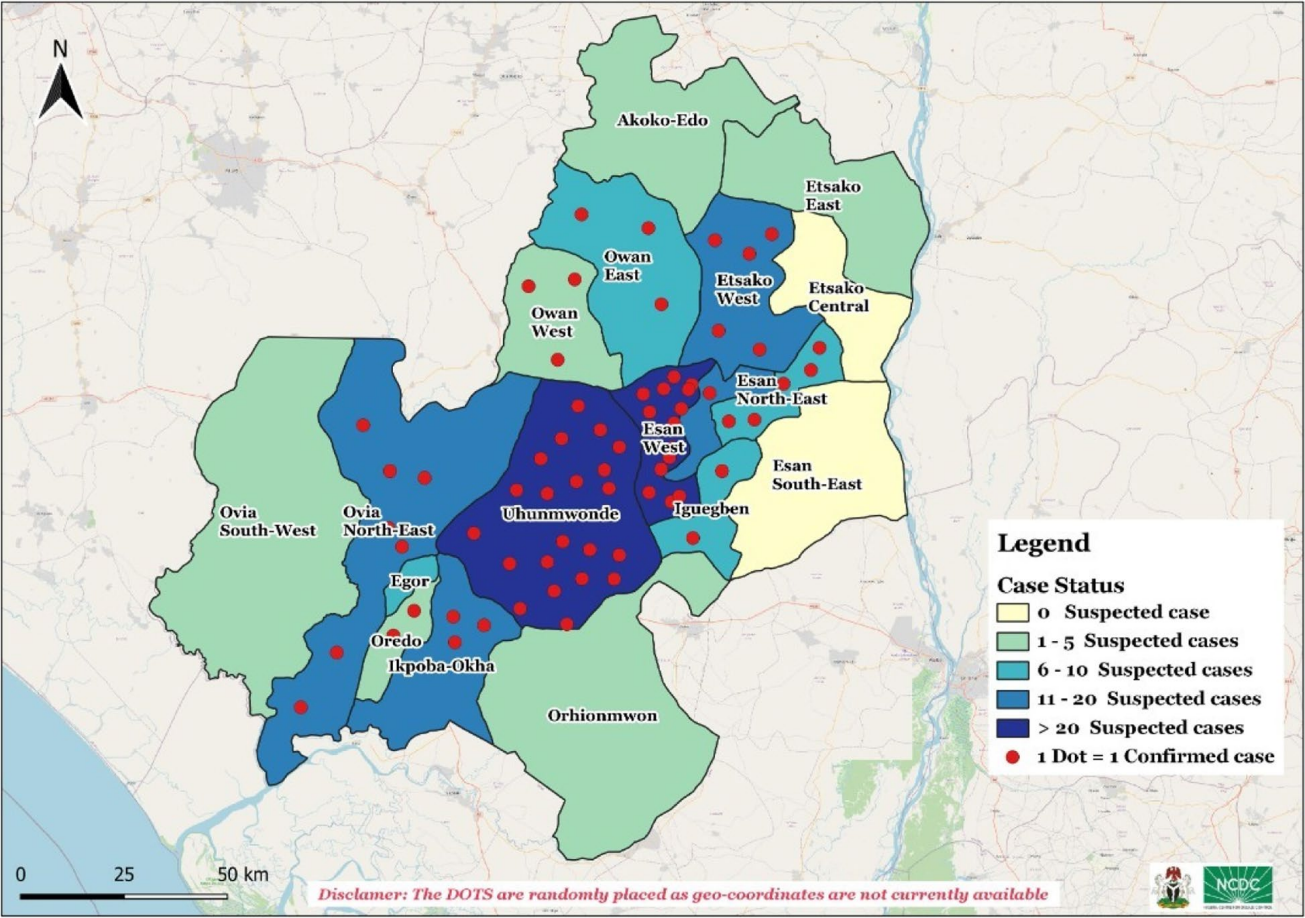
Map of Edo State showing distribution of suspected and confirmed yellow fever cases by LGA September 2018 – January 2019

The ages of the suspected cases were between 1 to 71 years [median: 20 years and range (70 years)], 159 (76.1%) were males and 50 (23.9%) females in a ratio of 3:1 (Table 1). The yellow fever outbreak affected more males within the age group of 11-30 years (Fig. 3). About 94 (45.0%) of the suspected cases were presumptive positive/inconclusive (IgM+) cases and 67 (32.1%) of Institut Pasteur Dakar confirmed cases were recorded. See Fig. 4 showing map of LGA distribution of cases. Blood samples of 174 (83%) cases were collected and sent to the laboratory. Twenty-five (12.0%) deaths were recorded from suspected cases, 17 (18.1%) deaths were recorded from presumptive positive cases (Table 1).

Among confirmed cases, the 67 (32.1%) confirmed cases, were reported from 12 LGAs. Male to female ratio is 3.2:1. Fifty-one (76.1%) were males; median age was 24.8, range 63 (1-64) years and 13 (19.4%) had history of YF vaccination. Fifteen deaths [Case fatality rate (CFR 22.4%)] were recorded.

**Fig. 5:**
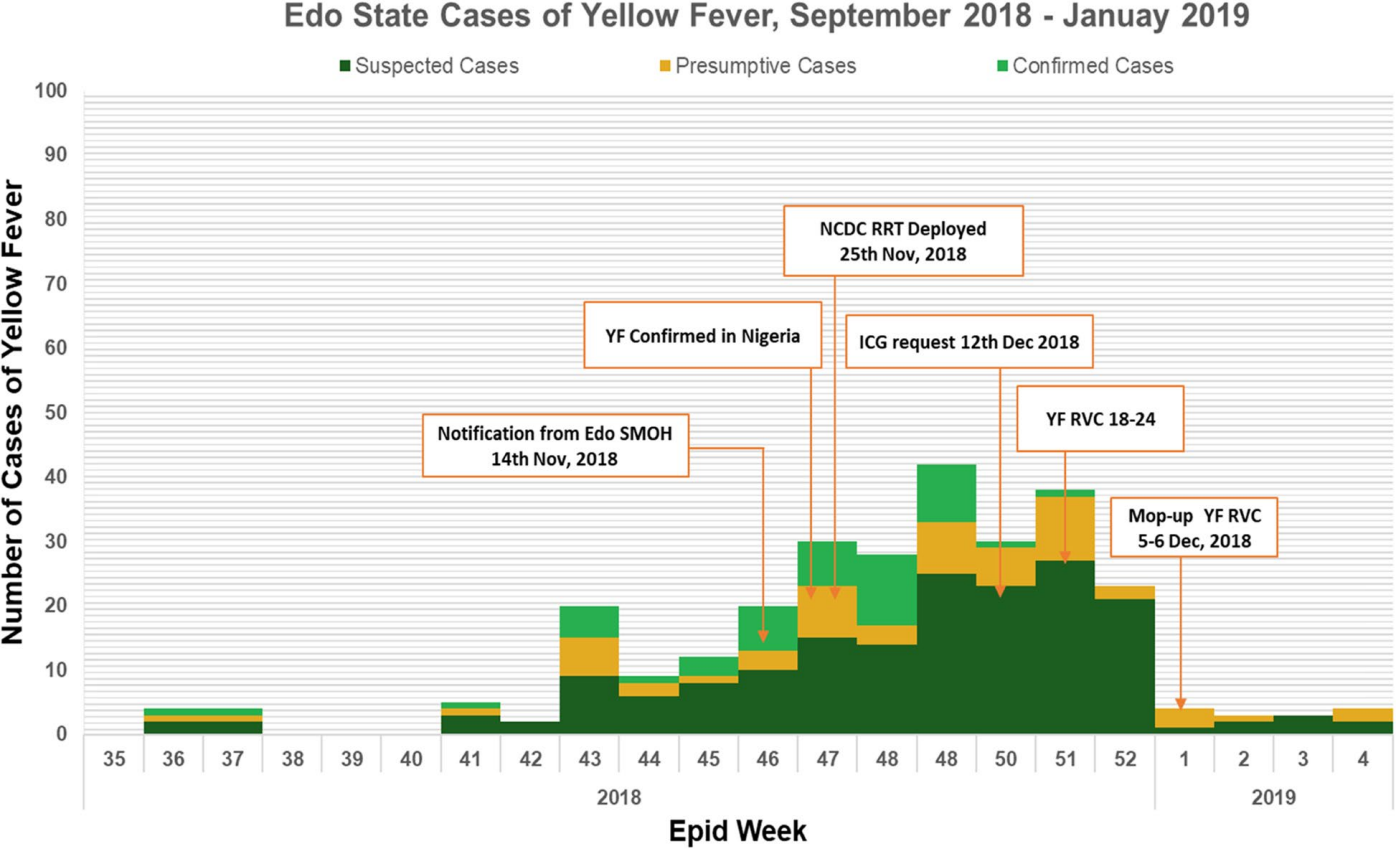
Epi curve of yellow fever cases in Edo State September 2018 - January 2019

Figure 5 shows the epicurve of the yellow fever outbreak and the timeline of response activities carried out during the outbreak. The height of the epicurve increased following enhanced active cases search which led to increase in case detection and the sharp drop in the epicurve following commencement of yellow fever vaccination in the affected LGAs. Yellow fever surveillance performance indicators were measured and compared with WHO standard. See Table 4.

Table four describes set of evaluating standards used to ensure that YF surveillance can meet the objectives of its surveillance system. About 6 (85.7%) of the performance indicators were achieved within the reporting period. However, 1 (14.3%), was lower than the target due to incomplete documentation of the date of release of laboratory result.

### Rapid vaccine coverage assessment

The yellow fever rapid vaccination coverage assessment was carried out in the first four LGAs that reported yellow fever cases. Two hundred and forty-one (241) children were assessed, targeting children aged 1 - 10 years [in Uhunmwode -178 (73.9%), Esan West - 47 (19.5%), Esan Central - 13 (5.4%), Ovia North-East - 3 (1.2%)]. Males accounted for 51.9% and females, 48.1%. Immunisation cards were available in 44.6% respondents and were completely filled in 33.7% of respondents. History of yellow fever vaccination was reported among 94 (39.0%). Yellow fever vaccination status among children that produced immunisation cards in all four affected LGAs was 81 (33.7%). When compared with other RI antigens, yellow fever vaccination had the lowest number of children vaccinated.

### Verbal autopsy

There were cases of deaths recorded from the communities in affected LGAs associated with yellow fever based on the case definition. Four deaths were recorded from two LGAs that were not captured by the state surveillance team; Uhunmwode (2), Ovia North-East (2) in which two were male and two females. All four cases presented with fever and jaundice before death.

### Summary of risk assessment

Table 5 presents the outcome of the risk assessment of yellow fever transmission conducted in Edo State during the outbreak response. Overall, the risk of yellow fever spread in the state was high (71.4%) based on the assessment criteria.

### Entomology survey

A total of 355 larval containers were inspected from 170 houses in 7 different settlements in the four LGAs in Edo state. Of these, 36.5% of the houses were positive for *Aedes* larvae while 28.5% of water retaining containers were positive for various immature stages of *Aedes* species. The immature stages of *Aedes* mosquitoes were collected mainly in abandoned domestic water containers and in leaf axils of banana plantation in all the LGAs sampled. Presence of vectors of yellow fever (*Ae.aegypti, Ae.albopictus* and *Ae.simpsoni*) were established in all LGAs sampled. High larval index was observed as shown by House and Breteau index. See Table 6.

### Reactive mass vaccination campaign

**Fig. 6:**
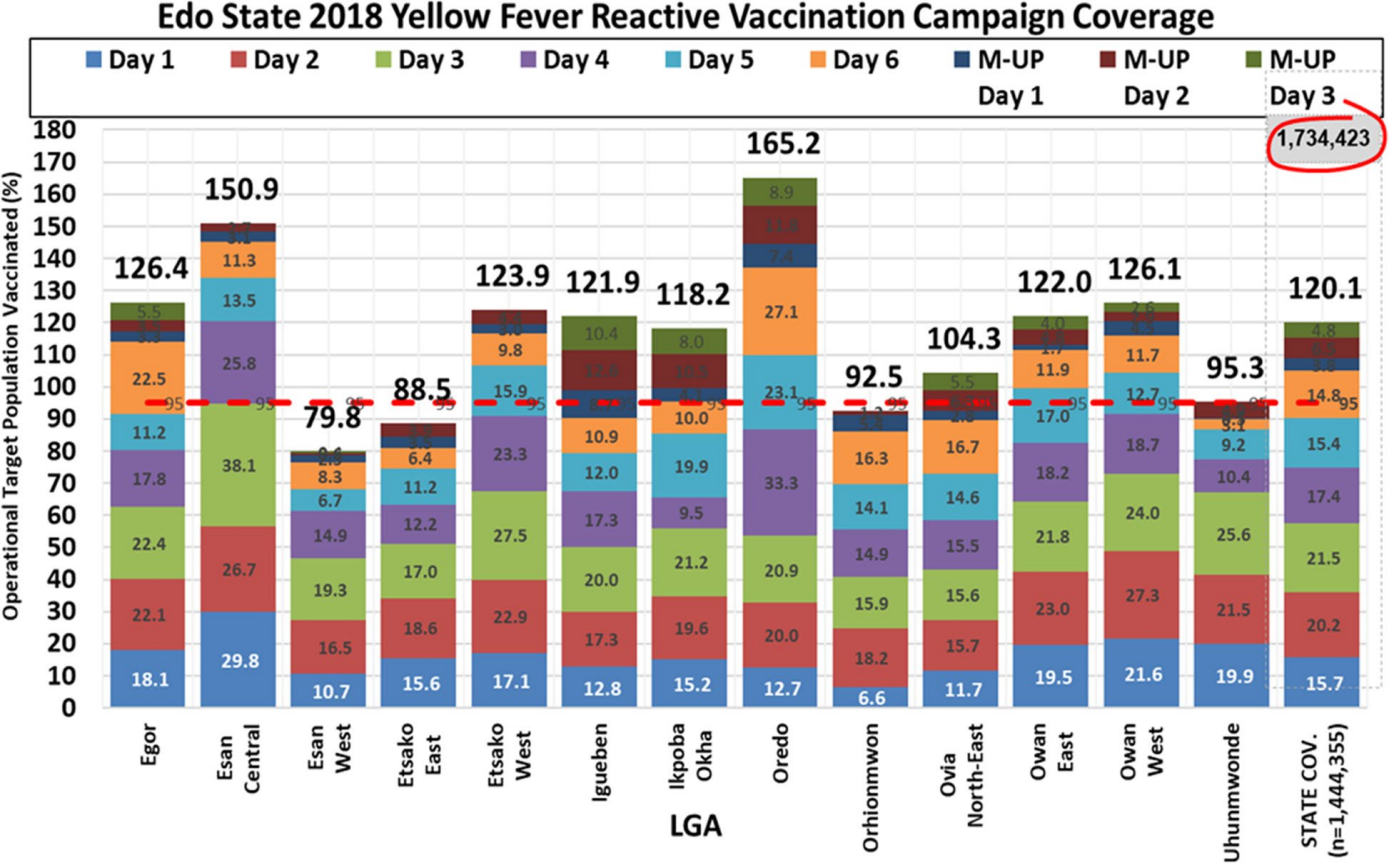
Yellow fever administrative coverage during yellow fever reactive mass vaccination campaign in Edo State December 2018-January 2019

Following the ICG request and approval on the December 12, 2018, reactive vaccination campaign was implemented in 13 (72.2%) of the 18 LGAs on December 18, 2019. These LGAs had confirmed yellow fever case(s) or contiguous with a LGA that had confirmed case. A total of 1,734,423 persons were vaccinated during the campaign in the 13 LGAs that implemented the yellow fever RMVC. Ten (76.9%) LGAs had administrative coverage above 95% benchmark needed to achieve herd immunity for yellow fever infection. See Fig. 6.

## Discussion

The Edo State YF outbreak is the most severe outbreak since the re-emergence of YF in Nigeria in 2017, defined in terms of number of cases, geographic spread, and mortality. The burden of the disease was higher than initially reported and the risk of transmission in the state was high as shown in the rapid risk assessment. In 2017, Kwara State had a similar occurrence of a YF outbreak [7, 20]. The transmission could be described to be of sylvatic origin because of the presence of forests and non-human primates (NHPs) which later spread to semi-urban and urban areas. We have observed that majority of yellow fever outbreaks in Nigeria could be of sylvatic transmission as observed in Kwara, Kogi and Zamfara and other affected states. However, the Edo State yellow fever outbreak transited from sylvatic to urban transmission.

An important consideration which influences the transition cycle from sylvatic to urban is internal migration. The most affected areas in Edo State are rural and peri-urban areas with distances between 40 km to 90 km to urban areas of Benin City, with substantial daily traffic. WHO has observed that with increased virus circulation and intense population migration from infected forest areas to urban settings, many large cities were burdened by yellow fever epidemics in West African countries and simultaneously, many smaller cities are still exposed to the disease [21]. This serves as a critical indicator to intensify efforts in controlling outbreaks in rural areas, to avoid wider urban yellow fever transmission which has more devastating effects.

There was a delay in reporting the outbreak due to poor index of suspicion of yellow fever among clinicians. Edo State is one of the states with the highest burden of Lassa fever in Nigeria [11]. This may have contributed to the delay in diagnosis as most of the initial YF cases had similar presentations with Lassa fever which has higher index of suspicion among clinicians in the state. Similar presentations were seen in Kwara [7] and Kogi yellow fever outbreaks. Again, with high burden of both Lassa fever and yellow fever in the state, the concept of co-circulation of multiple viral haemorrhagic fever (VHF) diseases needs further investigation [22]. This necessitates the need for training of health care providers on the epidemiology and surveillance of yellow fever at all levels of health care system to improve early detection and underscores the need for the introduction of multi-pathogen kits for the diagnosis of VHFs [23].

From our study, the male gender is more in number than the female and the predominant age group affected is 11 – 30 years. The gender and age groups implicated are the most productive and adventurous set in the population. They are more involved in out-door activities and occupations. Control programmes should target this population group.

Table 3 presented most of the symptoms observed among confirmed cases of yellow fever during this outbreak. In line with Simon, Hashmi and Torp observation, in their documentation on complications of YF, identified multiorgan failure, acute respiratory distress syndrome (ARDS), sepsis, respiratory failure, myocarditis, encephalitis, heamorrhage, disseminated intravascular coagulation (DIC) [24]. Most of our confirmed cases registered some of these complications.

**Table 3:** Presenting symptoms of confirmed cases of yellow fever in Edo State September 2018 – January 2019

Presenting symptoms among confirmed cases	Number of confirmed cases [(***n*** = 67), Percent (%)
Fever	40 (59.7)
Weakness	27 (40.3)
Jaundice	24 (35.8)
Bleeding	20 (29.9)
Vomiting	19 (28.4)
Head & Dizziness	15 (22.4)
Abdominal pain	13 (19.4)
Dehydration	12 (17.9)
Unconscious	9 (13.4)
Restlessness	9 (13.4)
Irrational talking	9 (13.4)
Poor appetite	5 (7.5)

**Table 4:** Summary of yellow fever performance indicators for surveillance in Edo State from September 2018–January 2019

Yellow fever performance indicators for surveillance	Maximum	Target (%)	Performance
Percentage of LGA reporting (Total number of LGAs in Edo =18 LGAs)	18	> 80	16 (88.9)
Percentage of LGAs that collected blood samples from at least one suspected case of yellow fever per year: target ≥80% (*n* = 16)	18	> 80	16 (88.9
Percentage of all suspect cases for which specimens were collected: target ≥50%. (*N* = 209)	209	≥50	189 (90.4)
Percentage of cases investigated within 48 hours of notification: target ≥80% (*n* = 189)	189	≥80	167 (88.4)
Percentage of samples sent to the laboratory within three days of investigation: target ≥80% (*n* = 189)	189	≥80	154 (81.5)
Percentage of samples reaching laboratory in adequate/good condition: target ≥80% (*n* = 189)	189	≥80	157 (83.1)
For IgM test: laboratory results reported < seven days after receipt of blood specimen: target ≥80% (*n* = 189)	189	≥80	82 (43.4)

**Table 5:** Rapid yellow fever risk assessment analysis for Edo State September 2018–January 2019

Description	Risk	Response	Score
Any suspected case(s)?	Yes	Yes	1
Any presumptive positive case(s)?	Yes	Yes	1
Any IP Dakar confirmed case(s)?	Yes	Yes	1
Sharing international borders?	Yes	No	0
Presence of International Airports in the state?	Yes	No	0
Sharing border with state(s) that have reported outbreak yellow fever?	Yes	Yes	1
Presence of any center/office for adult YF vaccination in the state?	No	Yes	0
^a^Any known forest in the state/presence of NHP?	Yes	Yes	1
City greater than 250,000 population in the state?	Yes	Yes	1
Any security compromised area in the state?	Yes	No	0
RRT Rapid YF vaccination coverage < 80%?	Yes	Yes	1
Any YF Campaign (PMVC or RVC) in the state (partial or total)?	No	No	1
Collected any species of Aedes mosquitoes?	Yes	Yes	1
Any case recoded from verbal autopsy?	Yes	Yes	1
**Total 14**			**10 (71.4%)**

^a^_*NHP* Non-human primates: The community members confirmed presence of NHP in Ehor Ward in Uhunmwode LGA_

**Table 6:** Determination of larval indices in entomological survey in Edo State September 2018–January 2019

Location	LGA	House index (%)	Container index (%)	Breteau Index
Ehor	Uhunmwode	10.0	3.3	10
Igieduma	Uhunmwode	53.3	38.1	106
Oke	Uhunmwode	33.3	15.0	10
Irue	Uhunmwode	26.7	20.3	50
Irruah (ISTH)	Esan central	50.0	35.7	75
Emaudo	Esan West	45.0	68.9	115
Ozogwo	Ovia North/East	60.0	75.0	120

High larval Index = House Index ≥5% and/or Breteau Index ≥20

Low larval Index = House Index < 5% and/or Breteau Index < 20

Case-based surveillance is an important strategy in the control of YF. It helps to identify high risk areas for preventive mass vaccination campaigns, promptly detects outbreaks for emergency vaccination response required by the IHR and must be reported to the WHO within 24 hours [25, 26]. There are targets set to monitor the efficiency and effectiveness of the yellow fever surveillance system. In this study, most of the surveillance performance monitoring indicators surpassed the target when compared with the Kwara State yellow fever outbreak [7]. Upon confirmation of yellow fever, a RRT was deployed to support the state with the response. There was intensive active case search, quick blood sample collection from active cases, improved communication, sample shipment and testing in the laboratories. Both national and state multi-agency public health Emergency Operation Centres (EOCs) were activated to effectively coordinate the responses. All these timely responses contributed to improve surveillance performance indicators for the outbreak.

The yellow fever vector which transmits the virus is the *Aedes* mosquito*.* They are diurnal (active during the day), bite both humans and animals alike, with early mornings and evenings marking their peak biting periods. *Aedes* species normally breed in transient water collections. These include collections in tree cavities, leaf axils, bamboo stumps, rock pools and artificial containers (including tin cans, coconut shells, domestic water storage containers, discarded vehicle tyres, broken earthen and ceramic wares). These breeding habitats were common in locations affected by yellow fever in Edo State and most of the larvae samples drawn from them were positive for different developmental stages of the Aedes mosquito, facilitating disease transmission.

Most of the Ovitraps set in some locations were positive for *Aedes* eggs. This is particularly significant and indicates high risk of transmission because infected *Aedes* mosquitoes are capable of spreading the yellow fever virus via transovarial transmission to the offspring [27] The risk of transmission is heightened by the fact that their eggs can remain viable for a maximum of 8 months [28].

The immature stages of *Aedes* mosquitoes were collected mainly in abandoned domestic water containers and in leaf axils of banana plantation in all the communities sampled. The findings from larval survey in all the locations where immature stages were collected revealed the presence of *Aedes albopictus and Aedes aegypti* which are both peri-domestic and domestic breeders and *Aedes simpsoni* a peri domestic breeder, commonly breeding in leaf axils around homes [29]. They are also known to be competent vectors for yellow fever virus [30]. The survey was conducted during dry season in which case most domestic and peri-domestic containers were without water, precipitating leaf axils to be the most common breeding sites. This is not unexpected as it is common for community members to surround their homes with banana plantations, the leaf axils of which contains water. The presence of canopy breeders whose active period is late at night was not established because of security reasons.

In our study, high larval index was observed with House Index ≥5% and/or Breteau Index ≥20 in all locations except in Ehor where the house index was low. This is a clear indication that all the communities are at high risk of yellow fever outbreak. The presence of yellow fever virus and competent vectors in the communities is a source of concern in Nigeria due to low vaccination coverage in the state; the proximity of a yellow fever cases to Benin City (an urban area in the state) and the potential spread to new LGAs and contiguous states in which case, all unimmunized individuals may be at high risk. We therefore advocate for enhanced YF vector surveillance and control.

The yellow fever rapid vaccination coverage was low in the LGAs visited and majority of the cases had zero dose vaccination status. This may account for the high number of cases with increased mortality due to low herd immunity for yellow fever in the state. Unlike the Kwara State outbreak that affected the nomadic population with known history of poor immunisation uptake [7], Edo State outbreak started among the indigenous population. Our findings revealed poor card retention for routine immunisation where only 81 (33.7%) of those with history of yellow fever vaccination could produce yellow fever vaccination cards. The implication of this is a gap in the routine immunisation services which needs to be strengthened. The poor yellow fever vaccination card retention among care-givers could account for the low vaccine coverage assessment observed.

The importance of effective leadership and communication during health emergencies was evident in this response. On notification of the outbreak to NCDC, there were timely coordinated response activities through the activation of Level Two Incident Management System (IMS) at both the national and state levels. Through the IMS, deployment of RRT, resource mobilisation and partners’ support were all intensified. Yellow fever blood sample management and transport were efficient and timely; request for yellow fever vaccines through the International Coordinating Group for vaccine provision (ICG) was successful with a swift approval coordinated by the National Primary Healthcare Development Agency (NPHCDA). Upon approval of the ICG request, available doses of yellow fever vaccines from other states that recently implemented YF preventive mass vaccination campaign were mopped-up before release and shipment of the ICG approved yellow fever vaccines to Nigeria. Consequently, the reactive mass vaccination campaign in the thirteen most affected/contiguous LGAs was implemented. And we recommended the provision of additional vaccines to support the implementation of YF vaccination campaign in the remaining 5 LGAs in accordance with the EYE Strategy [31, 32].

### Limitations of the study

Poor and incomplete documentation at heath facilities made active case search and line-list inclusion of some suspected cases cumbersome leading to exclusion of some suspected cases. In addition, some suspected cases may have been omitted from the line-list because of lack of collection of blood samples during active case search. The uncertainty of the level of security hindered some of the activities of the entomology team. For example, no sampling targeting the canopy breeders was done. This was because the peak activity of the canopy breeders is between 6:30 pm and 7:30 pm which was considered unsafe and hence the presence of canopy breeders could not be established.

The team could not complete the needed number of children per settlement in Ovia North-East because of other exigencies associated with the outbreak.

## Conclusions

Edo State had the highest burden of yellow fever cases in Nigeria since the 2017 when Nigeria recorded the first confirmed case of yellow fever after 21 years. More males were affected among the confirmed. Major symptoms include fever, jaundice, weakness, and bleeding. Majority of surveillance performance indicators were above WHO set target. There is a high risk of transmission of the disease in the state. Low yellow fever vaccination coverage, and presence of yellow fever vectors (*Ae.aegypti, Ae.albopictus* and *Ae.simpsoni*) were responsible for cases in affected communities. Enhanced surveillance, improved laboratory sample management, reactive mass vaccination campaign, improved yellow fever case management and increased risk communication/awareness were very important mitigation strategies to be sustained in Edo state to prevent further spread and mortality from yellow fever.

## Supplementary Information

**Additional file 1: Supplementary Fig. 1.** Suspected Yellow fever cases in Edo State by LGAs September 2018 – January 2019. **Supplementary Fig. 2.** Yellow fever attack rate by LGA in Edo State September 2018 - January 2019. **Supplementary Fig. 3.** Age-sex distribution of Yellow Fever cases in Edo State September 2018 - January 2019.

## Data Availability

All data sets analysed and included in this paper were based on experience(s) from the field during the outbreak response. In addition, the datasets used and analysed are available from the corresponding author on reasonable request.
