# Supplementary material for: Epidemiological description of and response to a large yellow fever outbreak in Edo state Nigeria, September 2018 - January 2019
Source: BMC Public Health. 2022 Aug 30;22:1644. doi: 10.1186/s12889-022-14043-6 (PMC9425801; doi:10.1186/s12889-022-14043-6)
Supplement: Supplementary file 1 — Additional file 1: Supplementary Fig. 1. Suspected Yellow fever cases in Edo State by LGAs September 2018 – January 2019. Supplementary Fig. 2. Yellow fever attack rate by LGA in Edo State September 2018 - January 2019. Supplementary Fig. 3. Age-sex distribution of Yellow Fever cases in Edo State September 2018 - January 2019. [file 12889_2022_14043_MOESM1_ESM.docx]

**List of Supplementary Figures for Edo State Yellow Fever Manuscript**

Supplementary Figure 1: Suspected Yellow fever cases in Edo State by LGAs September 2018 – January 2019

Supplementary Figure 2: Yellow fever attack rate by LGA in Edo State September 2018 - January 2019

Supplementary Figure 3: Age-sex distribution of Yellow Fever cases in Edo State September 2018 - January 2019
